# Bilateral Cellulitis Presented As Lower-Extremity Pain Reported in a Chiropractic Clinic: A Case Report

**DOI:** 10.7759/cureus.35470

**Published:** 2023-02-25

**Authors:** Eric Chun-Pu Chu, Colin Lai, Kevin S Huang, Andy Fu Chieh Lin

**Affiliations:** 1 Chiropractic Department, EC Healthcare, Hong Kong, HKG

**Keywords:** swelling ankle, ankle pain, cellulitis, chiropractor, chiropractic therapy

## Abstract

Bilateral lower-extremity cellulitis is a rare but serious condition that can lead to long-term health complications if left untreated. Herein, we report a case of a 71-year-old obese male with a two-month history of lower-extremity pain and ankle swelling. Magnetic resonance imaging (MRI) revealed the presence of bilateral lower-extremity cellulitis, which was confirmed through blood culture by the patient's family doctor. The patient's initial presentation of musculoskeletal pain, limited mobility, and other features coupled with MRI findings served as indications for timely referral to the patient's family doctor for further evaluation and management. Chiropractors should be aware of the warning signs of infection and the importance of advanced imaging for diagnosing such cases. Early detection and prompt referral to a family doctor for care can help prevent long-term health complications associated with lower-extremity cellulitis.

## Introduction

Bilateral lower-extremity cellulitis is a rare but serious condition that can lead to long-term health complications if left untreated [1]. The lower extremities are usually affected by cellulitis, which is an infection of the skin and subcutaneous tissues that is characterized by redness, pain, and swelling [2]. This condition can have a variety of underlying pathology, including venous eczema, lipodermatosclerosis, and open skin wounds [1]. Careful history collection and physical examination should be performed by a qualified medical professional to determine the underlying cause of the condition. Microbiological testing of the affected area may be necessary, although this is usually of minimal value in the absence of open skin wounds [3]. Treatment of bilateral lower-leg cellulitis typically involves antibiotics, drainage of abscesses, elevation of the affected area, and oral or topical anti-inflammatory medication. It is important to seek medical attention as soon as possible to avoid long-term complications and other medical conditions.

Chiropractors are healthcare professionals who are knowledgeable regarding the diagnosis and treatment of musculoskeletal conditions [4]. Symptoms of cellulitis can manifest as lower-extremity pain. Patients may not be aware of the underlying cause of their discomfort and may seek care from a chiropractor because chiropractic treatment rarely has any side effects [5]. Infectious cases are rarely reported in chiropractic centers; a previous study of 7,221 adults in Hong Kong revealed that only 0.04% of patients present with a new episode of lower-back pain [6]. Nonetheless, it is important for chiropractors to recognize signs of serious pathology and refer patients for appropriate medical attention to ensure the best possible outcomes.

Herein, we report the rare occurrence of bilateral cellulitis, which was the cause of lower-extremity pain, reported in a chiropractic clinic. We aimed to highlight the responsibility of chiropractors to be attentive in identifying such cases of bilateral cellulitis and referring those affected for the right medical care, as it is caused by a bacterial infection that can be difficult to treat without the help of antibiotics.

## Case presentation

A 71-year-old obese male experienced bilateral lower-extremity pain and ankle swelling for approximately two months. He reported feeling tightness and glossy stretching on the skin of his ankles, and the ankles showed redness in color. Swelling appeared after a day of walking with muscle aches and joint stiffness. Pain intensity was rated as 2/10 in the numeric pain score. Skin redness or inflammation increases after twisting the ankle for 24 hours; however, this patient reported no prior trauma or sprain. Additionally, the patient experienced fatigue and tenderness, particularly in the right ankle, as well as increased night urination frequency during sleep. The patient described that he sometimes had ankle sprain when walking in a hurry. Ankle radiography did not reveal any bony abnormalities. He was treated with a non-steroidal anti-inflammatory drug and was referred to the chiropractor for rehabilitation of the lower extremities.

He had a history of low-grade fever and intermittent diarrhea following coronavirus disease 2019 two months prior. He denied any history of dyspnea, chest pain, weariness, abdominal pain, night sweats, and hematochezia. He had chronic diabetes for 20 years and had been taking medication. He is a retired police officer who used to walk a lot and who also engages in social smoking and drinking. He had a family history of hypertension and diabetes with his father.

**Figure 1:**
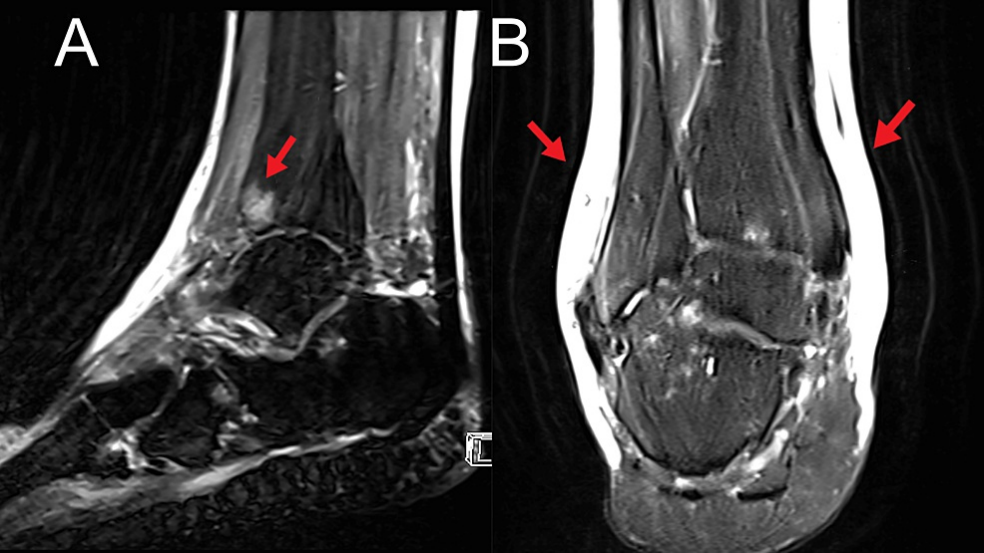
Right ankle magnetic resonance image. (A) On the sagittal image, subchondral marrow edema at the distal tibia and talus (arrow) was identified. (B) On the coronal image, diffuse subcutaneous soft tissue edema around the ankle is noted (arrows). The findings are suggestive of cellulitis.

**Figure 2:**
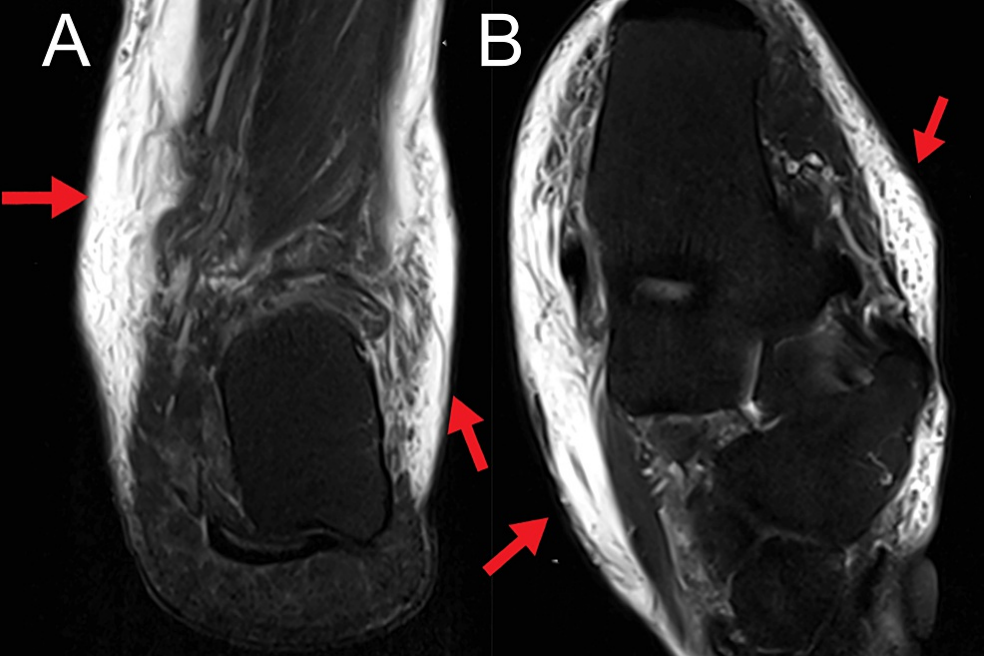
Left ankle magnetic resonance image. (A) On the coronal image, diffuse subcutaneous soft tissue edema around the ankle is noted (arrows). (B) On the axial image, subcutaneous soft tissue edema around the ankle is also noted (arrows). All findings are suggestive of cellulitis.

Physical examination revealed sclerotic skin changes in the plantar surface of the left foot accompanied by diffuse subcutaneous soft tissue edema around the ankle. The pitting edema test was performed by pressing a finger into the ankles, with indentation lasting 4 seconds on the right ankle and 2 seconds on the left ankle. A diminished sensation around the ankle joints and reduced motor strength in bilateral ankle inversion and eversion were noted. Orthopedic examination revealed reduced inversion and eversion motion of the right ankle with mild soreness when pressing the L5/S1 segments. Furthermore, the Thompson test and lumbosacral flexion and extension tests were both negative. All these findings indicated a sustained injury or trauma to the ankle and soft tissue around the ankle. To rule out structural pathology, the chiropractor advised bilateral ankle magnetic resonance imaging (MRI) to assess ankle pathophysiology, which revealed bilateral subcutaneous soft tissue edema around the ankles and soft tissue edema at the Kager’s fat pad (Figures 1A, 1B, 2A, 2B). On the basis of medical history, clinical presentation, and radiological results, the patient was diagnosed with cellulitis and immediately referred back to his family doctor.

The patient's symptoms were investigated by his family doctor, and blood tests and a liver function profile were performed, which indicated elevated inflammatory markers (c-reactive protein) and erythrocyte sedimentation rate. Blood culture revealed the presence of *Streptococcus pneumoniae*. The patient was treated with oral antibiotics, specifically dicloxacillin, for cellulitis and over-the-counter non-steroidal anti-inflammatory drugs (NSAIDs) (ibuprofen) to reduce pain and inflammation. A warm compress was also recommended to reduce swelling and aid in recovery. Additionally, the patient was directed to elevate his legs during night to help lower the pressure in the area's blood vessels and improve the blood flow. A compression wrap was also applied to the patient to further reduce swelling and improve blood flow.

Within a week, the patient experienced a dramatic reduction in swelling, and after a follow-up appointment on the 14th day, all symptoms related to his condition had completely recovered.

## Discussion

Despite the lack of defined diagnostic criteria and consensus regarding the diagnosis of cellulitis, clinicians should be aware of its signs during physical examination and history collection [7]. Symptoms such as pain and erythema, along with laboratory findings such as leukocytosis, may indicate cellulitis, especially if the individual has a history of trauma. Information comparing the relative prevalence of infected cellulitis and non-infectious cellulitis mimics is lacking in the literature [8]. Misdiagnosis of this condition is common. A study conducted in the United Kingdom found that 15 of 50 patients admitted with bilateral erythema of the legs were misdiagnosed as having cellulitis [9]. Similarly, another study conducted in the United States reported that 28% of patients were misdiagnosed [10]. Therefore, it is important for healthcare professionals to remain vigilant in assessment to ensure the accurate diagnosis and treatment of cellulitis.

Swabs from open skin wounds can sometimes identify the infectious entry points. However, microbiological analysis of the intact overlying skin is typically of limited benefit [11]. Both ultrasound and computed tomography are not reliable in diagnosing cellulitis [12]. Although MRI is rarely performed in a patient with cellulitis and the diagnosis is often made by performing physical examinations and medical history, MRI is still sensitive for identifying subcutaneous fluid collections and abscesses as well as differentiating cellulitis from necrotizing fasciitis and infected myositis. As a result, MRI is typically performed for suspected complications or to rule out alternate diagnoses in cases of an uncommon appearance rather than for the more common purpose of detecting cellulitis [12]. The present case was also accurately confirmed by the ankle MRI findings.

Old age, diabetes, and obesity are key risk factors for cellulitis [13]. Acute skin infections affecting the dermis and subcutaneous tissues are referred to as cellulitis. Erysipelas is a term used to describe a more superficial form of lymphatic cellulitis, which affects the face or extremities and is caused by a streptococcal infection. Wound infections and diabetic foot infections are distinct conditions. It is also more common in obese, sedentary patients with venous illness and worsens over time leading to hardening of the skin tissues [14]. Diagnosing bilateral lower-extremity cellulitis in a 71-year-old patient with a long history of diabetes and obesity might be difficult because of many common characteristics.

Bilateral lower-extremity cellulitis is a complex condition that requires careful follow-up to monitor disease progression and treatment response [15]. Clinicians should not automatically prescribe antibiotics to patients with bilateral lower-extremity swelling and redness, as the condition is also likely to be associated with other conditions such as dermatitis, varicose veins, or contact allergies [16]. Misdiagnosis of bilateral cellulitis can result in antibiotic overuse and unnecessary hospitalization of the patients [17]. Clinicians must collect a thorough medical history and conduct a comprehensive physical examination to distinguish bilateral cellulitis from its mimicking illnesses [18]. Treatment efficacy provides insight into the accuracy of the current diagnosis and may indicate the need for an alternative diagnosis if clinical signs are not consistent with expectations [18]. Our patient responded positively to antibiotic treatment, confirming the accuracy of the current diagnosis.

This study provides an important example of how challenging it can be to recognize an underlying infection in a patient with initial presentation of ankle pain. Although non-musculoskeletal conditions are rarely observed in chiropractic centers, cases involving infection and cardiovascular emergencies have been reported in the literature [19,20]. In the present case, a 71-year-old man with ankle pain was referred to a chiropractor who advised an MRI scan that revealed bilateral cellulitis. A family doctor eventually diagnosed the patient with an infection and his ankle swelling was found to be primarily caused by this infection. However, the patient's age, chronic diabetes, and obesity may also have played a role, making it difficult to differentiate between the two causes of his symptoms. This case highlights the need for clinicians to carefully consider all the potential causes when diagnosing patients with ankle complaints.

## Conclusions

This study describes an adult male with a chief complaints of bilateral lower-extremity pain and edema, who was referred to a chiropractor. The patient was diagnosed with cellulitis. Chiropractors should be aware of the potential for undiagnosed infection in patients with leg pain and must be able to recognize potential signs during initial history collection and physical examination. If infection is suspected, providers should consider requesting advanced imaging, such as MRI, and refer the patient to the family physician for prompt management.

## References

[1] Raff AB, Kroshinsky D (2016). Cellulitis: a review. JAMA.

[2] Lazzarini L, Conti E, Tositti G, de Lalla F (2005). Erysipelas and cellulitis: clinical and microbiological spectrum in an Italian tertiary care hospital. J Infect.

[3] Chuang YC, Liu PY, Lai KL, Tseng CH (2022). Bilateral lower limbs cellulitis: a narrative review of an overlooked clinical dilemma. Int J Gen Med.

[4] World Health Organization (2005). WHO guidelines on basic training and safety in chiropractic. https://apps.who.int/iris/handle/10665/43352.

[5] Chu EC, Trager RJ, Lee LY, Niazi IK (2023). A retrospective analysis of the incidence of severe adverse events among recipients of chiropractic spinal manipulative therapy. Sci Rep.

[6] Chu EC, Trager RJ (2022). Prevalence of serious pathology among adults with low back pain presenting for chiropractic care: a retrospective chart review of integrated clinics in Hong Kong. Med Sci Monit.

[7] Keller EC, Tomecki KJ, Alraies MC (2012). Distinguishing cellulitis from its mimics. Cleve Clin J Med.

[8] Nguyen SP (2023). A case of “bilateral cellulitis” of the lower extremities. J Hosp Med.

[9] Elwell R (2015). Developing a nurse-led 'red legs' service. Nurs Older People.

[10] David CV, Chira S, Eells SJ, Ladrigan M, Papier A, Miller LG, Craft N (2011). Diagnostic accuracy in patients admitted to hospitals with cellulitis. Dermatol Online J.

[11] Neill BC, Stoecker WV, Hassouneh R, Rajpara A, Aires DJ (2019). Cellulitis: a mnemonic to increase accuracy of cellulitis diagnosis. Dermatol Online J.

[12] Cranendonk DR, Lavrijsen AP, Prins JM, Wiersinga WJ (2017). Cellulitis: current insights into pathophysiology and clinical management. Neth J Med.

[13] Radswiki T, Carroll D, Knipe H (2011). Cellulitis. Radiopaedia.org.

[14] Cheong HS, Chang Y, Joo EJ, Cho A, Ryu S (2019). Metabolic obesity phenotypes and risk of cellulitis: a cohort study. J Clin Med.

[15] Kroshinsky D, Grossman ME, Fox LP (2007). Approach to the patient with presumed cellulitis. Semin Cutan Med Surg.

[16] Hirschmann JV, Raugi GJ (2012). Lower limb cellulitis and its mimics: part II. Conditions that simulate lower limb cellulitis. J Am Acad Dermatol.

[17] Weng QY, Raff AB, Cohen JM (2017). Costs and consequences associated with misdiagnosed lower extremity cellulitis. JAMA Dermatol.

[18] Hepburn MJ, Dooley DP, Ellis MW (2003). Alternative diagnoses that often mimic cellulitis. Am Fam Physician.

[19] Chu EC (2022). Large abdominal aortic aneurysm presented with concomitant acute lumbar disc herniation - a case report. J Med Life.

[20] Chu EC, Trager RJ, Chen AT, Shum JS (2022). A 60-year-old man with gingivitis and poorly controlled diabetes developing low back pain 1 week following recovery from COVID-19 diagnosed with spinal abscess due to Streptococcus oralis. Am J Case Rep.

